# Enhanced Catalytic Activity and Energy Savings with Ni-Zn-Mo Ionic Activators for Hydrogen Evolution in Alkaline Electrolysis

**DOI:** 10.3390/ma16155268

**Published:** 2023-07-27

**Authors:** Ivana Perović, Milica Marčeta Kaninski, Gvozden Tasić, Sladjana Maslovara, Petar Laušević, Mina Seović, Vladimir Nikolić

**Affiliations:** 1 Department of Physical Chemistry, “Vinča” Institute of Nuclear Sciences—National Institute of the Republic of Serbia, University of Belgrade, Mike Petrovića Alasa 12-14, 11351 Belgrade, Serbia; gvozdentasic@vin.bg.ac.rs (G.T.); petarl@vin.bg.ac.rs (P.L.); mina050@vin.bg.ac.rs (M.S.); 2 Institute of General and Physical Chemistry, Studentski trg 12/V, 11158 Belgrade, Serbia; smaslovara@iofh.bg.ac.rs (S.M.); vnikolic@iofh.bg.ac.rs (V.N.)

**Keywords:** hydrogen evolution reaction, alkaline electrolysis, energy saving, ionic activators, catalysis

## Abstract

Green hydrogen produced by alkaline electrolysis is a promising solution to address the world’s increasing energy demand while mitigating greenhouse gas emissions. However, the efficient and cost-effective production of green hydrogen via alkaline electrolysis requires improvements. This paper presents an in situ activation process that simplifies the alkaline electrolysis technology while enhancing the catalytic activity of electrodes for the hydrogen evolution reaction. The aim of this research is to enhance the energy efficiency of alkaline electrolysis and decrease the energy consumption for hydrogen production. To achieve this goal, ionic activators comprising Ni, Zn, and Mo were incorporated into the standard electrolyte solution. Our results demonstrate that the anticipated improvement in the catalytic activity of the d-metal combination, surpassing even that of precious metals, has been successfully attained. As a result, a 20% reduction in energy consumption (REC) for the hydrogen produced has been observed. The catalytic activity of the added activators for the hydrogen evolution reaction was discussed by analyzing the mechanism of the reaction via Tafel analysis and EIS techniques. These findings offer a promising approach to improve alkaline electrolysis and enhance the production of green hydrogen.

## 1. Introduction

The efficient storage of energy obtained from intermittent renewable energy sources is crucial for improving these technologies. Storing surplus energy during periods of peak production guarantees a steady supply during times when renewable sources are inaccessible [1]. The most promising solution to this problem is the hydrogen economy, the backbone of which is hydrogen and hydrogen energy. The hydrogen economy is the most viable solution to this issue, as it is founded on hydrogen and its energy. The idea of a hydrogen-based economy was formulated as an alternative to fossil fuel-based economies as early as 1975 [2], but significant research toward improving technologies related to this concept has only been conducted in the last two decades.

The effective implementation of the hydrogen-based economy is heavily dependent on the cost of green hydrogen in the global market [3]. The production of hydrogen that is cost-efficient, energy-efficient, and has a low carbon footprint is crucial to meet market demands. To achieve this, reducing the energy losses of the hydrogen production process is key [4]. Recent progress in electrolysis technology has led to enhanced energy efficiency of the process by decreasing the initial production expenses and minimizing energy consumption and loss [5,6]. Any advancements in improving the efficiency of the electrolytic process hold great significance both scientifically and practically.

The electrolysis of water is a crucial method for hydrogen production, as it yields highly pure hydrogen that can be directly utilized in applications requiring high-purity hydrogen, such as fuel cells, without the need for additional purification steps. Moreover, when renewable energy sources like solar, wind, or hydro power are employed for the electrolysis process, it can significantly reduce or even eliminate environmental pollution.

There are various approaches to enhance the effectiveness of splitting water molecules into their constituents during the electrolysis process. These methods mainly involve improving the technical aspects of the electrolyzer, such as the geometry of the electrolytic cell, as well as developing new membranes, electrodes, and electrocatalytic materials [7]. This paper focuses on exploring the catalytic activity of different metals for the hydrogen evolution reaction (HER) in the context of the hydrogen evolution reaction. The catalytic activity of diverse metals is a periodic function of the atomic number, which is confined to the three periods of transition elements. The modulation in current density is indicative of the corresponding catalytic activity. The larger the exchange current density, the faster the reaction, and vice versa. Values of exchange current densities for HER as a function of the metal–hydrogen bonding energy (“volcano” curve) give us an insight into which metals are most suitable for the catalysis of the hydrogen evolution reaction. Considering that noble metals are at the top of the “volcano” curve, due to their expensive exploitation and application, researchers shifted their focus on finding new materials that will have similar or equal catalytic activity as noble ones, and at the same time, their application will be significantly more economical and available [8]. Brewer theory of intermetallic bonds tells us that the best ways to improve the efficiency of the electrolytic hydrogen evolution process is the presence of d-metals [9,10]. It predicts a synergistic effect of d-elements with hypo- and hyper-electronic configuration. The pairing of these elements creates a new phase that occupies some intermediate electronic configuration and is comparable to the configuration of individual transition metals. Taking this into account, as well as the fact that d orbitals participate in the process of adsorption of the reaction intermediate (for HER it is H_ads_), it is expected that alloys of two or more elements from the opposite sides of the “volcano” curve show a higher activity than they would individually. In some cases, it is possible that the activity of such alloys exceeds the activity of precious metals. The real application of this synergistic effect in electrolysis is based on the modification of the surfaces of nickel or stainless-steel electrodes with transition metal alloys. The method by which these modifications are achieved is the most common electrochemical deposition of transition metals from appropriate electrodeposition baths [11,12,13]. Recently, a new method is in use, which involves the direct addition of metals (so-called ionic activators, i.a.) to the electrolyte during the electrolysis process itself [14]. This method is called in situ ionic activation. It implies the presence of the ionic form of the d-metal complex in the electrolyte solution, which reduces the overvoltage for hydrogen extraction and enables a more energetically favorable transfer of protons from the electrolyte to the cathode surface.

In our previous studies, we have substantiated the exceptional in situ catalytic efficacy exhibited by different combinations of d-metals for the hydrogen evolution reaction. Particularly noteworthy among these combinations is the Ni-Co-Mo system [15]. Building upon these findings, the purpose of the present investigation is to explore the catalytic performance of a Ni-Zn-Mo combination of d-metals. This choice stems from the fact that zinc (Zn) is a more accessible and cost-effective element compared to cobalt (Co), rendering it highly significant for potential industrial applications. The HER mechanism was analyzed under various experimental conditions, including different temperatures and applied current densities. Additionally, the study examined the relationship between the concentration of ionic activators, operating temperature, exchange current density, and energy consumption during in situ activation.

## 2. Materials and Methods

The experiments were conducted in a 6M (30 wt%) KOH solution (Merck KGaA, Darmstadt, Germany, p.a.) that was prepared using deionized water (with a resistivity of 18 MΩcm^−1^). Salts and complex used as ionic activators: Sodium molybdate dihydrate (Na_2_MoO_4_·2H_2_O), Zinc chloride (ZnCl_2_) and Tris(ethylenediamine)nickel(II) chloride dihydrate ([Ni(en)_3_]Cl_2_·2H_2_O) were all ACS reagents (Merck KGaA, Darmstadt, Germany). Table 1 shows the composition of the electrolytes that were used in the experiments that investigated the influence of ionic activators in detail. According to Brewers theory of synergistic effect [16] of d-metals and based on experience from our previous research [17,18], we tested different ratios of Ni and Zn with constant concentration of the Mo salt. These concentrations are optimized for our experimental setup. The active surfaces of all electrodes used in this work were made of high-purity nickel (99.99%). Preceding each series of measurements, the electrodes were subjected to a rigorous cleaning procedure, involving polishing with 800 and 2000 grit polishing paper, which was followed by immersion in a HNO_3_ aqueous solution (molar ratio of 2:1) for a duration of 2 min and subsequent rinsing with deionized water and ethanol. The reference electrode was an SCE electrode, while a Pt mesh with a surface area considerably larger than that of the working electrodes under scrutiny was utilized as the counter electrode.

To investigate the impact of Ni-Zn-Mo activators on the kinetics of the hydrogen reaction, polarization curves were recorded using a three-compartment standard electrochemical cell. The current density range investigated was from 10 to 100 mA cm^−2^. The electrode potential was altered in the range of −1.15 to −0.75 V, relative to the SCE reference electrode, at a rate of 1 mVs^−1^. To correct for the potential drop across the electrolyte, the “current interrupt” method was employed. The recording of impedance spectra was performed in the frequency range from 0.1 to 100 kHz, using an alternating signal of amplitude 10 mV superimposed on a constant value of overvoltage in the range from −20 to −250 mV. Preceding every measurement, a constant potential was applied to the working electrode for a duration of 15 s to condition it. The temperature of the electrolyte solution within the electrochemical cell ranged between 298 and 343 K. Prior to each measurement, the electrolyte solution was thoroughly purged with hydrogen by continuous bubbling for a duration of 30 min. Due to the rapid kinetics associated with the hydrogen evolution reaction, it is imperative to conduct preliminary measurements following hydrogen bubbling. This precaution ensures that the evolution of hydrogen has a negligible impact on the local concentration, particularly in comparison to other gases such as nitrogen or argon. The Gamry Reference 3000 Potentiostat/Galvanostat/ZRA instrument (Gamry Instruments, Warminster, PA, USA) was employed to conduct all electrochemical measurements (quasi-potentiostatic and galvanostatic). Energy-efficiency measurements were performed in a monopolar electrolytic cell, which was described in our previous publication [19].

The water manometer apparatus, as described in our prior work [20], was employed to quantify the evolution time of hydrogen and oxygen (at a mole ratio of 2:1). The cathode morphology prior to and post-electrolytic process was investigated through scanning electron microscopy (SEM) analysis. The SEM images of the cathodes subjected to testing were obtained using JEOL—JSM—6610 (Jeol, Akishima, Japan) and Vega TS 5130MM (TESCAN, Brno, Czech Republic) instruments under high vacuum conditions and an accelerating voltage of 20 kV. The angle of inclination of the sample was confined between 0° and 30°, with the applied magnification being up to 3000×. The corresponding EDS spectra were captured utilizing the same instruments. XRF measurements were performed using the Thermo Scientific Niton XL3t Goldd+ XRF analyzer (Waltham, MA, USA) at room temperature.

## 3. Results and Discussions

**Figure 1:**
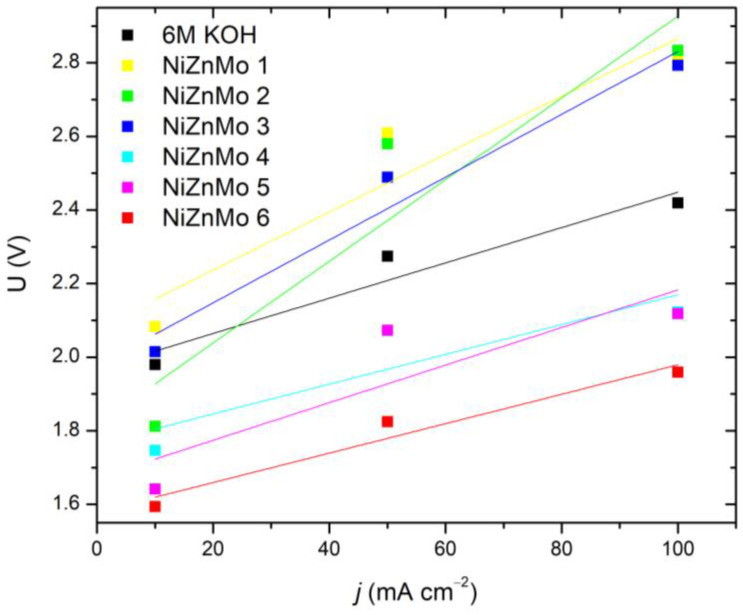
Voltage vs. current density of varying concentrations of the in situ added Ni-Zn-Mo ionic activators.

To begin with, the study aimed to identify the most favorable electrolyte composition for in situ ionic activation. Table 2 and Figure 1 illustrate the voltage dependence on various Ni-Zn-Mo ion activator concentrations and applied current densities. The data indicate that combination (6) exhibited the most favorable outcomes in terms of reduced voltage and energy consumption, in comparison to the baseline electrolyte, 6 M KOH. The most significant reduction in voltage occurred at the highest current density tested, which was 100 mAcm^−2^.

Detailed tests of energy consumption and reaction kinetics in alkaline electrolysis of in situ added Ni-Zn-Mo ionic activators were performed using the sixth combination of the mentioned activators (Ni-Zn-Mo (6)).

### 3.1. The Energy Efficiency of Water Electrolysis with In Situ Activation

The study investigated the relationship between energy consumption, temperature, and current densities in both a standard electrolyte (6 M KOH) and an electrolyte with the in situ addition of ionic activators Ni-Zn-Mo (6). The energy balance of electrolysis is represented by the energy consumed per mole of hydrogen produced, which was calculated on the basis of Formula (1):

*Q* = *U*·*I*·*t*, (1)

where *Q* is the energy consumption in J mol^−1^, *U* is the total voltage of electrolysis in V, i.e., the potential difference of the cathodic and anodic reactions and the voltage drop through the electrolyte, *I* is the total current in A, and *t* is the time in seconds for which 1 mol of hydrogen is developed.

**Figure 2:**
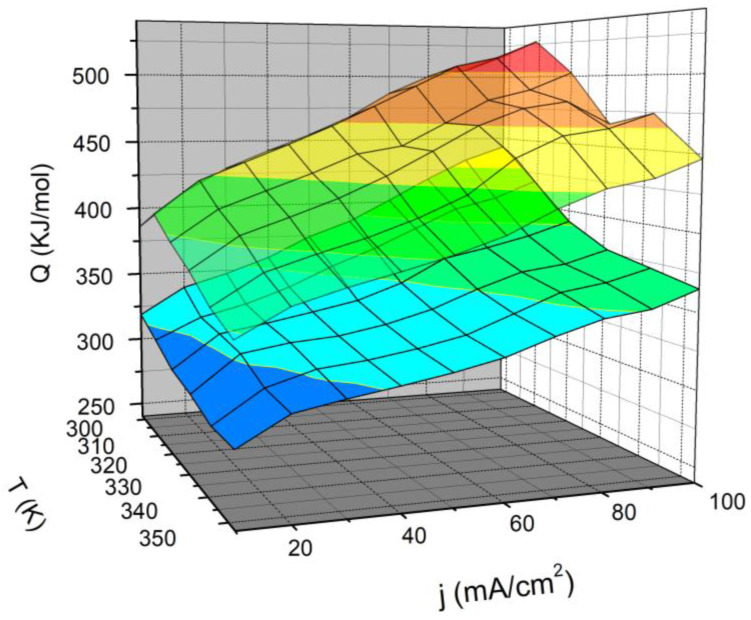
The relationship between energy consumption, temperature, and current density for the standard electrolyte (6 M KOH)—top surface, and with the in situ addition of the Ni-Zn-Mo ionic activator—bottom surface.

The results obtained from the analysis of this system are represented by a three-dimensional diagram in Figure 2 and numerically in Table 3.

The presented diagram indicates that higher temperatures result in greater energy savings both for the standard electrolyte and the electrolyte with added ionic activators. The improvement of the electrolytic process is affected by both the elevated temperature and the addition of ionic activators. The obtained data suggest that this system has promising potential for industrial application, as even at slightly lower temperatures, the energy savings exceed 20%, significantly reducing the price of hydrogen production. The reduction in energy consumption in this system can be explained by the theory of hypo-hyper-d-inter-electronic interactions, as mentioned earlier in the introduction [21,22]. It has been concluded that electrode coatings with the appropriate composition and structure of d-metals exhibit higher activity compared to individual metals and in some cases even higher than noble metals [16,23].

Earlier works investigating the influence of in situ added Ni(en)_3_Cl_2_ and molybdates as ionic activators [24,25,26] show that the combination of these two d-metals significantly increases the active surface area of cathodes. Additionally, the catalytic activity of ethylene-di-amine (en), present in the electrolyte after the decomposition of the cobalt complex, plays a significant role in electrode activation. This role can be described by the Roland effect, a phenomenon observed in the electrochemical reduction of cobalt complexes, where the addition of ethylene-di-amine (en) to the electrolyte enhances the catalytic activity of cobalt toward the reduction in substrates [27,28]. This effect cleans the electrode surface by removing metal oxides [29]. Overall, the catalytic activity of ethylene-di-amine is a result of its ability to stabilize and activate the intermediate species formed during the reduction process of cobalt complexes. The formation of nickel and molybdenum deposits is promoted on the cleaned electrode surface, which in the deposition process, it undergoes the same cleaning process due to the formation of ethylene-di-amine ligands during the process. These findings support the creation of a deposit that has a highly active surface for the hydrogen evolution reaction.

### 3.2. The Influence of In Situ Activation on the Rate and Mechanism of the Hydrogen Evolution Reaction

The kinetics and mechanism of the hydrogen evolution reaction in systems with in situ added ionic activators were investigated by the methods of Tafel analysis and EIS spectroscopy. Polarization curves and EIS spectra were recorded for standard electrolyte solution (6 M KOH) and electrolyte with in situ added ionic activators based on Ni-Zn-Mo (6).

#### 3.2.1. Tafel Analysis

**Figure 3:**
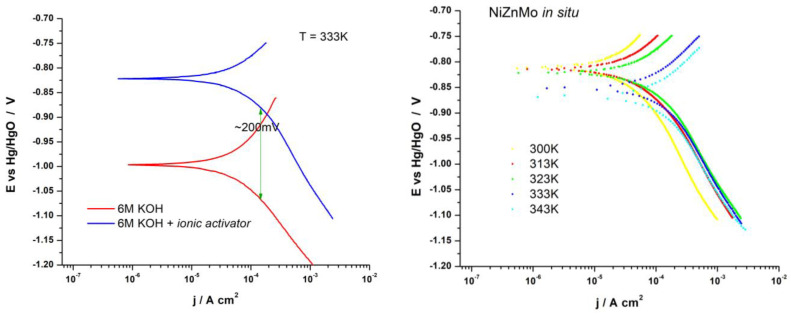
Polarization curves for HER in 6 M KOH with and without ionic activators (**left**) and polarization curves for HER with a Ni-Zn-Mo ionic activator added in situ in 6M KOH at temperatures from 300 to 343 K (**right**).

Polarization curves for the hydrogen evolution reaction recorded for a system with a pure nickel electrode in a standard alkaline electrolyte and in an alkaline electrolyte with added ion activators are shown in Figure 3. It is observed that in situ activation results in a shift of the electrode potential of a significant 200 mV toward more positive values in a wide range of potentials. The right part of Figure 3 shows the influence of temperature on the appearance of the polarization curve for in situ activated nickel electrodes.

The basic kinetic parameters, *j*_0_ and *b*, were determined by analyzing the linear part of the semilogarithmic polarization curves and presented in Table 4. The values of the Tafel slope of all recorded polarization curves are around −120 mVdec^−1^ and indicate that charge transfer (Volmer) is a slow and decisive step in the hydrogen evolution reaction. Based on the approximately equal slopes of the polarization curves both in the case of the standard electrolyte and the addition of the ionic activator, it can be concluded that in this case, the addition of Ni-Zn-Mo did not change the decisive step of the reaction, which indicates that the addition of ionic activators did not affect the change in the reaction mechanism of hydrogen evolution.

The exchange current density increases with temperature, which is the expected kinetic behavior for the observed reaction. The coating based on Ni-Zn-Mo electrochemically deposited at a temperature of 343 K, during the electrolytic process, shows the lowest values of overvoltage in the tested range of current densities and the highest value of the exchange current density, which confirms that this coating is catalytically much more active than the pure Ni electrode.

The mechanism of improving the kinetics of the electrode reaction by adding the Ni-Zn-Mo activator in situ can be described by the formation of a more developed electrode surface with a much larger number of active centers for hydrogen adsorption.

#### 3.2.2. Electrochemical Impedance Spectroscopy

**Figure 4:**
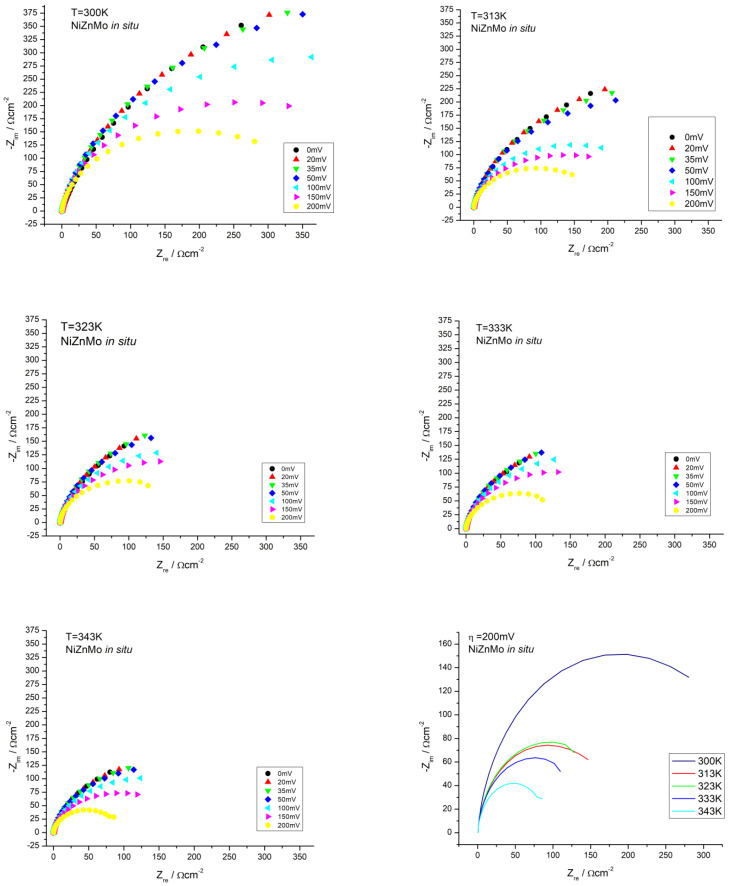
Nyquist plots of impedance spectra of the EIS in situ activation procedure with ionic activators based on Ni-Zn-Mo at various overvoltages and temperatures.

A better insight into the mechanism of the electrochemical reaction of hydrogen evolution was made possible by recording electrochemical impedance spectra for a pure nickel electrode in a standard electrolyte solution (6 M KOH) and for an electrolyte with an added Ni-Zn-Mo ionic activator. EIS spectra were recorded at overvoltages from 20 to 200 mV at temperatures from 298 to 343 K. Before each measurement, the electrodes were conditioned at a voltage of 1.3 V with respect to the SCE reference electrode for 15 s. Figure 4 shows the impedance diagrams (Nyquist diagrams) of the nickel working electrode in the electrolyte with an in situ added ionic activator.

The obtained data were fitted according to the Randels equivalent circuit model in which the capacity of the double electric layer was replaced by a constant phase element (CPE) and shown in Table 5.

The results presented in this study demonstrate that an increase in the applied overvoltage leads to a significant change in the impedance response and a decrease in the charge transfer resistance, *R_ct_*, thereby indicating an enhancement in the kinetics of the hydrogen evolution reaction. At all tested temperatures, a decrease in the value of the capacity of the double electric layer, *C_dl_*, can be observed with an increase in overvoltage. This can be attributed to the occlusion of the pores caused by the intense hydrogen bubble generation at higher overvoltages. At the same overvoltages, there is a noticeable increase in the value of the double electric layer capacity with increasing temperature. This is expected, considering that at higher temperatures, the resistance to charge transfer decreases. The influence of the surface on the catalytic activity can be determined by determining the roughness factor σ, which is directly related to the capacity of the electric double layer, *C_dl_*. After the electrolytic process and deposition of the coating based on Ni-Zn-Mo, the surface of the electrode is no longer smooth, so an increase in roughness is expected. When *C_dl_* exhibits purely capacitive behavior, the surface roughness can be estimated by assuming a certain value for a unit surface. In the case of metals, a typical value of *C_dl_* is around 20 mF cm^−2^ [30]. The roughness factors (σ) were then calculated by dividing the *C_dl_* value of the characterized electrode (presented in the Table 5) by that of the reference metal electrode. The obtained roughness factor (σ) values for the Ni-Zn-Mo-based ionic activators (i.a.) were found to be consistently high across all overpotentials and temperatures in comparison to pure nickel electrodes [31]. These results indicate that the improved electrocatalytic activity observed for the hydrogen evolution reaction (HER) using Ni-Zn-Mo-based i.a. is partially attributed to the increased active surface area.

#### 3.2.3. Elemental Composition and Morphological Characteristics

**Figure 5:**
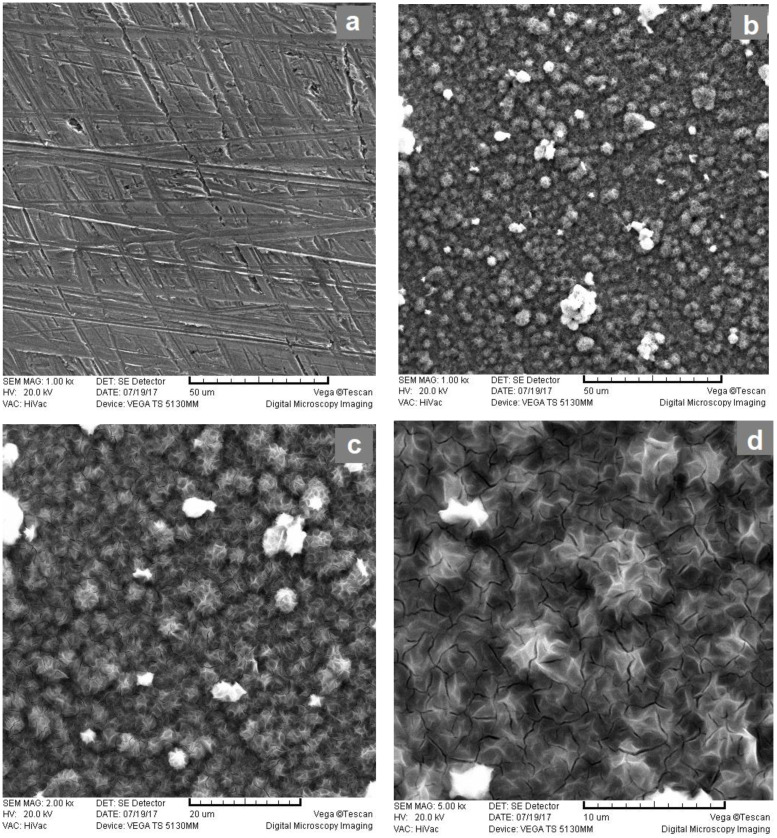
SEM images of: pure nickel cathode (**a**); after electrolytic process with Ni-Zn-Mo ionic activator at 1000× (**b**), 2000× (**c**) and 5000× (**d**) magnification.

The elemental composition and morphological characteristics of the nickel cathode before and after the electrolytic process with in situ added activators based on Ni-Zn-Mo were investigated using scanning electron microscopy (SEM) and XRF analysis. The obtained results are shown in Figure 5 and in Table 6.

Presented SEM micrographs show that there is a deposition of spherical structures of nanometer dimensions evenly distributed over the entire surface of the cathode. The surface of the cathode formed by the deposition of active species after the electrolytic process is much more developed compared to the surface of a pure nickel electrode. As a result, the number of active centers for the adsorption of intermediates of the hydrogen evolution reaction is significantly increased. This confirms the conclusions reached on the basis of electrochemical measurements.

The elemental composition of the resulting deposit, determined by the XRF analysis method, shows that the deposited particles are composed mostly of Ni. Zn and Mo are present in a much smaller percentage. The coating composition obtained by EDS analysis shows good agreement with the XRF analysis except in the case of nickel. This discrepancy can be attributed to the difference in the recording depth of the composite by these two methods and to the fact that the EDS analysis can see both the oxygen content resulting from exposure of the electrodes to air and the carbon content originating from the sample carrier in the SEM chamber. Given that nickel and zinc in the electrolyte are in cationic form, and molybdenum is in anionic form, it can be concluded that the induced co-deposition of these three metals occurred but to a lesser extent. Organic compounds like tris(ethylenediamine)nickel(II) chloride or dissolved gases in the electrolyte can undergo oxidation or reduction reactions, leading to the deposition of C or O onto the electrode surface. The presence of C species can be attributed to the chemical reaction involving tris(ethylenediamine)nickel(II) chloride. Furthermore, exposure of the electrode to atmospheric oxygen, in combination with the presence of sodium molybdate and tris(ethylenediamine)nickel(II) chloride in the electrolyte, can contribute to the oxidation process and explain the presence of O species. It is important to emphasize that the presence of C and O in the coating does not necessarily imply a detrimental effect on its performance.

## 4. Conclusions

The research findings demonstrate the superior catalytic activity of the investigated materials for the hydrogen evolution reaction compared to commercially available options. The utilization of in situ ionic activators composed of Ni-Zn-Mo can result in a notable decrease in energy consumption of up to 20% compared to a system utilizing pure nickel electrodes and a standard electrolyte solution. The observed decrease in energy consumption is pronounced at higher temperatures, whereas increasing current density is linked to Increased energy consumption. The mechanism behind the enhanced energy efficiency with the addition of ionic activators can be attributed to several interrelated effects that are involved in the overall process of in situ activation of alkaline electrolysis. One such effect is the catalytic effect, which is a synergistic consequence of the combination of transition metals. Additionally, the surface effect, which is a result of the simultaneous co-deposition of the catalytic coating and hydrogen evolution, is a critical factor. This process leads to the formation of a highly developed surface coating with numerous active centers for hydrogen adsorption. The contribution of the ethylene-di-amine ligand cannot be disregarded, as its impact is comparable to that of ethylene-di-amine tetra acetic acid (EDTA), which removes oxide layers from the cathode surface, thereby facilitating better surface development and metal deposition.

In the investigation of electrode kinetics, it was observed that the electrode activity exhibited a consistent trend of improvement, as was also noted in the energy consumption measurement experiments. Upon employing in situ activation across all tested current ranges, a marked reduction in overvoltage for the hydrogen evolution reaction was observed when compared to non-activated pure nickel electrodes operating in a standard electrolyte solution.

The investigation into the mechanism of the hydrogen evolution reaction, which was obtained by determining the values of the Tafel slopes, indicates that the slow step of the reaction in the tested system of ionic activators Ni-Zn-Mo is charge transfer, i.e., Vollmer’s reaction step.

Furthermore, during the in situ activation, in addition to the hydrogen evolution reaction, a parallel process of electrochemical deposition of the alloy of d-metals is present on the cathode surface, which is easily observed based on SEM images of the formed coatings and their EDS and XRF analysis for all tested systems.

The results presented in this study demonstrate that the application of in situ ionic activators consisting of Ni-Zn-Mo can yield a substantial reduction in energy consumption and enhance electrode kinetics. This approach shows promise in advancing alkaline electrolysis and promoting the production of green hydrogen.

## Figures and Tables

**Table 1:** Composition of investigated electrolytes with ionic activators.

Solution No.	*c*(KOH)/M	*c*([Ni(en)_3_]Cl_2_·2H_2_O)/M	*c*(ZnCl_2_)/M	*c*(Na_2_MoO_4_·2H_2_O)/M
1	6	1 × 10^−3^	1 × 10^−3^	1 × 10^−2^
2	6	1 × 10^−3^	5 × 10^−3^	1 × 10^−2^
3	6	1 × 10^−3^	1 × 10^−2^	1 × 10^−2^
4	6	5 × 10^−3^	1 × 10^−3^	1 × 10^−2^
5	6	1 × 10^−2^	1 × 10^−3^	1 × 10^−2^
6	6	5 × 10^−2^	1 × 10^−3^	1 × 10^−2^

**Table 2:** Voltage–current density relationship at 298 K for varying concentrations of the ionic activator.

*j* (mA cm^−2^)	*U* (V)						
	**6 M KOH**	**Ni-Zn-Mo**					
		**(1)**	**(2)**	**(3)**	**(4)**	**(5)**	**(6)**
10	1.980	2.083	1.812	2.015	1.747	1.642	**1.594**
50	2.274	2.609	2.58	2.489	2.072	2.073	**1.825**
100	2.419	2.806	2.834	2.793	2.123	2.118	**1.959**

**Table 3:** Alkaline electrolyzer energy consumption at higher current densities and at higher temperatures.

	***Q*/kJmol^−1^**					
	**6 M KOH**			**Ni-Zn-Mo i.a.**		
	**T/K**			**T/K**		
***j*/mAcm^−2^**	**303**	**323**	**333**	**303**	**323**	**333**
**60**	477.2	448.4	423.1	369.6	341.0	333.0
**70**	492.3	465.1	445.9	384.2	353.3	345.3
**80**	507.4	476.9	459.8	398.3	363.7	357.7
**90**	514.2	481.5	464.4	405.9	368.2	362.8
**100**	527.1	465.9	474.3	418.4	377.0	371.0

**Table 4:** Kinetic parameters for Ni electrodes in 6M KOH and upon addition of Ni-Zn-Mo activator.

*T*/K	-*b*/V dec^−1^		*j_0_*/A cm^−2^	
	*i.a.*	6 M KOH	*i.a.*	6 M KOH
300	0.131	0.131	20.6 × 10^−6^	23.95 × 10^−6^
313	0.129	0.128	36.48 × 10^−6^	30.38 × 10^−6^
323	0.119	0.126	46.71 × 10^−6^	39.66 × 10^−6^
333	0.114	0.112	64.17 × 10^−6^	58.16 × 10^−6^
343	0.115	0.095	69.04 × 10^−6^	63.15 × 10^−6^

**Table 5:** Parameters obtained by fitting experimental EIS spectra recorded at different values of overvoltage and temperature for tested Ni-Zn-Mo coatings.

*T* (K)	−*η* (mV)	*R_e_* (Ω cm^2^)	*R_ct_* (Ω cm^2^)	*C_dl_* (mF cm^−2^)	τ/s	*σ*
**300**	20	0.305	990.8	2.830	2.804	141
	35	0.338	909.1	2.429	2.208	121
	50	0.340	863.0	2.215	1.911	110
	100	0.342	646.1	1.980	1.279	99
	150	0.342	463.0	1.834	0.849	91
	200	0.342	344.1	1.791	0.616	89
	250	0.305	990.8	2.830	2.804	141
**313**	20	0.238	510.9	3.904	1.994	195
	35	0.237	480.5	3.638	1.748	181
	50	0.237	439.6	3.478	1.528	173
	100	0.238	266.3	3.290	0.876	164
	150	0.238	222.2	3.351	0.744	167
	200	0.238	169.5	3.187	0.540	159
	250	0.238	510.9	3.904	1.994	195
**323**	20	0.222	476.7	8.181	3.900	409
	35	0.224	460.3	7.240	3.332	362
	50	0.224	419.3	6.739	2.825	336
	100	0.226	306.8	6.142	1.884	307
	150	0.228	252.7	5.467	1.381	273
	200	0.228	174.9	4.874	0.852	243
	250	0.222	476.7	8.181	3.900	409
**333**	20	0.212	392.9	9.922	3.898	496
	35	0.214	381.3	8.745	3.334	437
	50	0.215	362.8	7.926	2.875	396
	100	0.213	280.6	6.528	1.831	326
	150	0.209	223.1	5.570	1.242	278
	250	0.209	140.9	5.016	0.,706	250
**343**	20	0.215	301.2	9.148	2.755	457
	35	0.212	287.1	7.762	2.228	388
	50	0.212	267.6	6.999	1.873	349
	100	0.209	215.6	6.060	1.306	303
	150	0.205	161.9	5.365	0.868	268
	200	0.203	93.9	4.777	0.448	238

**Table 6:** Elemental composition of Ni-Zn-Mo coating recorded by EDS and XRF analysis.

Element	Ni	Zn	Mo	C	O
**EDS**	23.62%	1.21%	0.19%	32.86%	40.83%
**XRF**	98.1%	1.4%	0.2%	/	/

## Data Availability

Not applicable.
